# Toward Robust Non-Intrusive Load Monitoring via Probability Model Framed Ensemble Method

**DOI:** 10.3390/s21217272

**Published:** 2021-11-01

**Authors:** Yu Liu, Yan Wang, Yu Hong, Qianyun Shi, Shan Gao, Xueliang Huang

**Affiliations:** 1 School of Electrical Engineering, Southeast University, Nanjing 210096, China; 220202976@seu.edu.cn (Y.W.); 220203012@seu.edu.cn (Q.S.); shangao@seu.edu.cn (S.G.); xlhuang@seu.edu.cn (X.H.); 2 Jiangsu Provincial Key Laboratory of Smart Grid Technology and Equipment, Nanjing 210018, China; 3 State Grid Lianyungang Power Supply Company, Lianyungang 222000, China; hongyu@js.sgcc.com.cn

**Keywords:** dictionary learning, ensemble method, non-intrusive load monitoring, probability model, uncertainty analysis

## Abstract

As a pivotal technological foundation for smart home implementation, non-intrusive load monitoring is emerging as a widely recognized and popular technology to replace the sensors or sockets networks for the detailed household appliance monitoring. In this paper, a probability model framed ensemble method is proposed for the target of robust appliance monitoring. Firstly, the non-intrusive load disaggregation-oriented ensemble architecture is presented. Then, dictionary learning model is utilized to formulate the individual classifier, while the sparse coding-based approach is capable of providing multiple solutions under greedy mechanism. Furthermore, a fully probabilistic model is established for combined classifier, where the candidate solutions are all labelled with probability scores and evaluated via two-stage decision-making. The proposed method is tested on both low-voltage network simulator platform and field measurement datasets, and the results show that the proposed ensemble method always guarantees an enhancement on the performance of non-intrusive load disaggregation. Besides, the proposed approach shows high flexibility and scalability in classification model selection. Therefore, by initializing the architecture and approach of ensemble method-based NILM, this work plays a pioneer role in using ensemble method to improve the robustness and reliability of non-intrusive appliance monitoring.

## 1. Introduction

### 1.1. Background

With the fast development of whole society, energy awareness of people is gradually growing. Such trend is conducive to dealing with both energy and climate crises, and therefore strongly supported by academic groups and industrial institutions [1]. As the main energy consumption form, electric power is highly regarded due to its wide tight connections with end users. Hence, it is valuable to take full advantage of power data to mine energy use patterns, provide energy consumption insights and contribute to the energy saving implementations [2].

Under such a background, smart socket is proposed at the early stage to capture the energy patterns of specific appliance as needed [3]. Usually, the smart socket is deployed to the plug of target appliance, and utilized for the monitoring of only certain appliance. In addition to the signal acquisition and processing module, the communication module is also required in smart socket [4]. Therefore, multiple appliances monitoring requires multiple sockets, and it is really expensive to deploy the smart socket-based sensing networks in a house. Besides, by allocating the sockets at the plug of each target appliance, it is, in essence, an intrusive load monitoring (ILM) approach, which is commonly not welcomed by household occupants [5].

Considering the disadvantages and limitations of the socket-based monitoring approach, non-intrusive load monitoring technology is emerging as a promising household energy use monitoring solution in recent years [6]. Proposed by Professor Hart from MIT, non-intrusive load monitoring (NILM) aims at monitoring the individual appliance in a non-intrusive way, i.e., decomposing the integral power consumption data measured at the service panel into individual appliance power use profiles [7]. Obviously, several advantages appeal by implementing such non-intrusive strategy. Since decomposition algorithms are utilized to replace the measurement hardware, while the power meter can directly provide the integral signals from service panel, no additional devices are required. Therefore, economic benefits have been obtained, along with the friendly experience that the occupants are not interfered [8]. Acting as the virtual sensing networks in household energy use monitoring, NILM is highly welcomed by all participants and highly expected in advanced management of future home energy systems [9].

Recently, another appliance monitoring scheme, namely semi-intrusive load monitoring (SILM), has also been proposed and investigated [10]. Basically, the key idea of SILM is to find a trade-off between hardware costs and monitoring performance by partitioning appliances into blocks and disaggregating the detailed consumption of each block via separate smart meters [11]. Apparently, compared with NILM, the addition of smart meters would enhance the monitoring performance, but also require higher investments. So, major research on SILM focus on the optimal allocations of additional meters [11] as well as the economic analysis [10]. However, in each meter monitoring block, the detailed appliance behaviors are also monitored through NILM techniques. So, the emergence of SILM furtherly boost the research and development of NILM. Since the robust and reliable load disaggregation is the precondition of the NILM applications, the major focus at the current stage is still on how to improve the monitoring performance.

Technically speaking, the most effective ideas to improve the monitoring performance can be categorized into following two aspects, i.e., utilizing adequate load signatures or applying efficient disaggregation algorithms. Real power and reactive power are the most commonly used signatures, which have been discussed in the original study [7]. Once different types of home appliances are proved to have diverse harmonic features [12], the current harmonics are considered as a powerful signature to identify different appliances in NILM. The selections of the order and number of harmonics for appliance identification are investigated in [13], where odd order harmonics are highlighted and different minimal subsets of harmonics may be recommended by diverse heuristic strategies. Other load signatures, such as V-I trajectory [14] and phase noise [15], are also investigated. However, the more complicated the signatures, the higher the sampling rate. Therefore, the real power, reactive power and harmonics are the most popular signatures from the view of practical implementations, and the required sampling rate is related to the need of harmonic order. Besides, these steady state characteristics can be organized in low frequency data form, as discussed in [16], which is easy to store and transfer.

As to the disaggregation algorithms, the non-intrusive load disaggregation follows the typical optimization idea, that the problem can be described as selecting the optimal combination of electrical appliances to match the integral signals. Therefore, mathematical optimization model has been utilized for NILM since the early days, and also acts as an important solution in recent researches [17]. However, because the low calculation efficiency and optimality of results cannot be guaranteed by optimization algorithms, limitations are shown for the NILM problems. As the researches go further, researchers gradually find that the essence of NILM problems matches the pattern recognition well, so the related methods are widely investigated. Clustering algorithm has been utilized in the early days [7], and still regarded as a significant solution to residential household load monitoring problem, e.g., k-means clustering [18] and c-means-based approach [19]. Bayes classifier has also been proved to be an efficient energy disaggregation approach under non-intrusive strategy [20], and further supports the unsupervised non-parametric modeling with other appliance operation patterns, such as time of usage [21]. Support vector machine (SVM), another typical pattern recognition approach, has been explored for the NILM problem, even for addressing the unknown appliances [22]. Demonstrated to be effective, investigating NILM algorithms based on pattern recognition emerge as a hot topic in academic field, and various approaches including principal component analysis (PCA) [23], non-negative matrix factorization (NMF) [24], and Gaussian mixture model (GMM) [25] are all verified to be contributions.

### 1.2. Research Gap

Above investigations on load signatures and disaggregation algorithms have shown some achievements in NILM problems under certain conditions. However, in the practical situations, it is not feasible to make all the measurements or data comply with the specific sampling requirements. The actual practical implementation is to develop a robust solution, which is able to guarantee or improve the disaggregation performance with the given dataset. Toward this goal, researchers have tried to combine diverse approaches together to achieve a more robust and effective solution considering the physical features of NILM problem [19,24,25]. Our team tried to utilize the advantages of optimization method, i.e., using particle swarm optimization (PSO) to act as an auxiliary means of the whole solution and finally contribute to the NILM performance enhancement, in [26]. The idea of integrating different approaches together is indeed effective in improving the load disaggregation performance, and it exactly matches the principle of ensemble methods. Ensemble methods show a giant superiority in various artificial intelligence competitions, and is considered as the most potential representative solution in the future machine learning and deep leaning fields [27]. Due to the strong potentials, ensemble methods have already been introduced and utilized in power systems analysis, such as renewable power forecasting [28] and security assessment [29], and proven superior compared with traditional machine learning approaches [30].

According to the fundamental principles and mechanisms of ensemble methods, it is possible to improve NILM performance for the given measurements or data, no matter which specific disaggregation algorithm is applied. The key is the effective design of the ensemble strategy, where the reliability and variability of individual classifiers matter. Researchers from New Zealand have conducted some preliminary explorations by using ensemble machine learning techniques in NILM [31]. Although only water heating is considered and the ensemble design is constrained to the problem scenario, which highly limits the potentials for widespread applications, such an attempt is an encouraging exploration. Nevertheless, to our knowledge, the ensemble method-based NILM research are relatively scarce at the present stage, and the reason is mainly due to the high difficulty of ensemble scheme design. In other word, the successful implementation of ensemble methods requires that the individual classifiers are reliable and differentiated, which is hardly guaranteed under traditional deterministic framework of NILM.

### 1.3. New Contributions

As known, the decision making of ensemble methods is based on the outputs of individual classifiers and evaluation of combined classifier. Under traditional framework, the individual classifier is a complete NILM approach and outputs a specific solution. Such mechanism can hardly guarantee the reliability and variability of multiple individual classifiers at the same time, especially the variability requirement. In order to design a proper ensemble model, a lot of efforts are required to allocate the individual classifiers, as well as the decision process of combined classifier. Such attempts are with high difficulty and failure rates. So, although ensemble method has been demonstrated to be effective in NILM problem [31], the explorations of related studies are limited.

However, the essence of the NILM problem is the uncertainty analysis, i.e., determining the best fittings of the target signals with the maximum probabilistic likelihood. Though rarely referred, this probabilistic description has been hidden in most NILM studies. Our work in [32] has provided the illustration of the probabilistic decision-making process in the problem of non-intrusive energy use monitoring for a group of electrical appliances, revealing the uncertain nature of NILM. Following the design of probabilistic models, the output of individual classifiers is no longer constrained to specific solution, but can be generalized to a set of solutions labelled with probabilities. Such change makes the output of individual classifiers more multifarious, which copes with the diversity requirement for individual classifiers and leads to the effective ensemble strategy. Therefore, the idea of introducing the probability quantization makes the ensemble method-based NILM formulation easier to design, which is an easy and feasible strategy to explore the ensemble method embedded NILM.

Based on above observations, we establish a universal ensemble method framework for NILM in this paper, which is featured by probabilistic quantization and easy-to-design characteristic. At first stage, the ensemble framework is featured for NILM problem, while the feasibility is guaranteed by probability modelled modules and the scalability is achieved by flexible selection and allocation of individual classifiers. Then, a probability model framed ensemble method design, exampled by dictionary learning [33], is illustrated. Based on the greedy characteristics of dictionary learning, a probabilistic quantitative scoring system is established for evaluation of the multiple candidate solutions of individual classifier. A two-stage campaign approach for decision-making is embedded with combined classifier, for the purpose of outputting reliable disaggregation results. Finally, the proposed idea and method are tested on both low voltage network simulator platform and field measurement datasets, and the results verify the effectiveness and superiority of this work. By providing an easy-to-design ensemble framework and demonstrating its feasibility and superiority for NILM problems, more related studies may be inspired in non-intrusive sensing field.

The major contribution of this paper is the proposal of the easy-to-design ensemble method-based NILM framework, which is featured with probabilistic models. It is a reliable approach to enhance the NILM performance, with flexibility and scalability. The detailed contributions can be summarized as follows.

- A universal and easy-to-design NILM framework based on ensemble method is established, where the probability model is introduced to guarantee the effectiveness of ensemble strategy.
- The individual classifier is modelled by dictionary learning, where multiple candidate solutions are outputted under greedy mechanism.
- A quantitative evaluation approach based on the probabilistic scoring system is proposed, to measure the likelihood of each candidate in a fair way.
- The combined classifier is modelled by a two-stage decision-making process, while the first stage is based on voting and the second stage is based on scoring.
- The proposed framework and method are verified by both simulation platform and field measurement datasets, proving the effectiveness and superiority of utilizing ensemble method in NILM performance enhancement.

The rest of this paper is organized as follows. Section 2 provides the problem statement, where the proposed framework of ensemble method-based NILM is given. Dictionary learning-based formulation and probabilistic decision-making are illustrated in Section 3. Results are discussed in Section 4 and conclusions are drawn in Section 5.

## 2. Problem Statement

From the view of physical point, NILM is a problem that identifying the individual appliances from the integral power signals. However, when abstracted as a scientific question, it is indeed a classification problem in the field of pattern recognition [34]. Recent scientific research and practical experiences show that the ensemble strategy is the most effective way to maximize the advantages and benefits of individual classifier, and finally output the best results in artificial intelligence learning process [35]. Therefore, the framework of ensemble method is presented and revised to cope with the features of NILM problem in this section.

### 2.1. NILM Problem Formulation

In our work, the load disaggregation is considered from the view of steady state operations, i.e., disaggregating the steady state running signals rather than events. Under the non-intrusive load monitoring framework, the disaggregated appliances can be determined by the best fitting of the measured integral steady state signals. The disaggregation could be challenging since the operation modes of some appliances are complicated and there may be background noise in practical scenarios. To be specific, the electrical features, such as real power, reactive power and harmonics, should be matched correspondingly.

(1) $$\left.\begin{array}{c} P=\sum P_{i}\\ Q=\sum Q_{i}\\ H=\sum H_{i} \end{array}\right\}i\in \Omega$$

where *P*, *Q*, and *H* are the integral signal vectors of real power, reactive power and current harmonics, respectively. *P_i_*, *Q_i_*, and *H_i_* are the *i*th appliance selective energy signatures, and are in the same length with *P*, *Q*, and *H*. Ω stands for the candidate appliance set, where all target appliances are included. In order to acquire the desired harmonics, the sampling rate should satisfy the Nyquist-Shannon sampling theorem. Our proposed approach should be able to enhance the NILM performance based on given power signals, i.e., no matter which power signatures are utilized. The Equation (1) illustrates a complete model, but the proposed approach should also be effective if some power signatures are lacking.

### 2.2. Ensemble Method Framework for NILM

**Figure 1:**
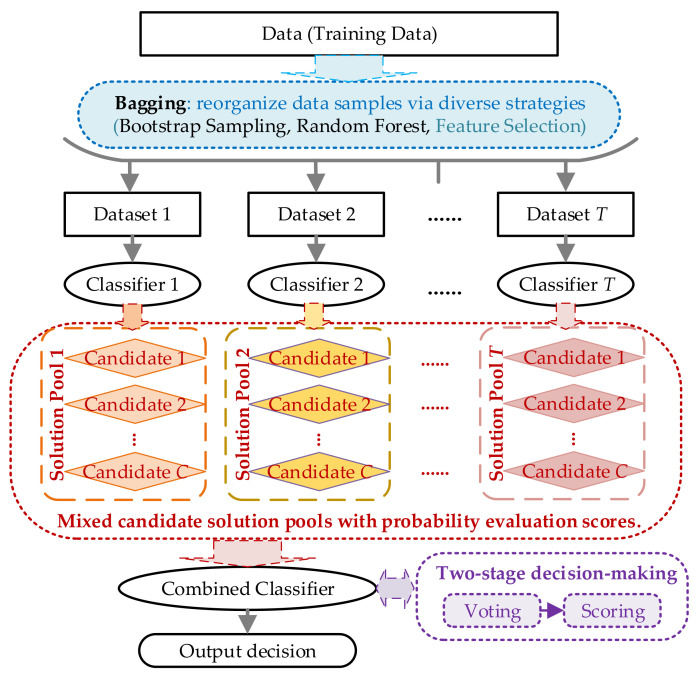
Proposed framework of non-intrusive load monitoring (NILM)-oriented ensemble method.

At current stage, the most powerful approach dealing with the classification problem in (1) is ensemble method, whose framework is shown in Figure 1. Notice that the modules in black blocks represent the traditional structures of ensemble approach, while the colored parts are our proposed improvements to take the characteristics of NILM into consideration.

As seen in Figure 1, the ensemble framework is following the bagging strategy, and also featured and revised with major NILM characteristics. The featured enhancements can be summarized as follows.

- Bagging in blue color. Bagging method is critical for the final performance of ensemble approaches. Traditionally, bootstrap sampling and random forest are the two commonly used approaches for bagging. Considering the diversity of sampling data in NILM problem, the electric power signatures can also be combined for data resampling. Note that no matter which strategy is utilized, the individual classifier should be mutually divergent with reliable performance.
- Solution pools in red color. This part is customized for the NILM problem, and also the key of our enhancement. Back to the essence of NILM, it is a fitting estimation problem. If each individual classifier could output a set of solutions, the real solution is more likely to be included compared with conventional single result. Therefore, by establishing the solution pool for individual classifier, it increases the possibility of the real solution locating among the candidates. Of course, for a scientific judgement for all candidates, a fair evaluation approach is required. Here the probabilistic evaluation scoring based on distance measures is utilized.
- Decision-making in purple color. In order to accurately recognize the real solution in all candidates, a two-stage decision-making strategy is proposed. The basic idea is to apply qualitative voting in the first stage to narrow down the range of candidate solutions, and then use quantitative scoring to find the real solution. Voting in first stage exactly matches the decision-making principle of original ensemble method, and scoring in second stage follows the probability model established in the proposed framework. So, this strategy fully combines the characteristics of both problem and method, which highly copes with our expectations.

Following the framework shown in Figure 1, a new train of thought solving the NILM problem is presented. For a better illustration and demonstration, the detailed methodologies and solutions are exampled and discussed in the following sections. Nevertheless, it’s worth noting that the proposed framework has good versatility and flexibility, which is not limited to the following implementations.

## 3. Methodology

The key points to successfully implement the proposed ensemble scheme are the valid selections of individual classifiers and combined classifier. In this section, the detailed strategy and model to allocate the classifiers are given firstly, followed by the algorithm flow dealing with NILM.

### 3.1. Dictionary Learning for Individual Classifiers

As mentioned above, there are two requirements for individual classifiers, i.e., distinction and reliability. Bootstrap sampling and random forest are the conventional methods to guarantee the distinction [36], which is not introduced here but will be discussed in the case section. The feature selection approach, which matches the NILM characteristics, will be presented and extended in detail here. As to the reliability, a credible load disaggregation model based on dictionary learning is utilized [33].

#### 3.1.1. Dictionary Learning Model

Since the NILM tries to disaggregate the integral signals into appliance components, it matches the idea of dictionary learning well. Given a target signal *x*∈*R^S^*^×1^, if we have a dictionary *D* = [*d*_1_,*d*_2_,…,*d_N_*]∈*R^S^*^×*N*^, whose column *d_k_*∈*R^S^*^×1^ is defined as atom, then the dictionary learning model is established by the following linear combination.

(2) x=D⋅α

where *α*∈*R^N^*^×1^ is defined as sparsity parameter.

Obviously, the sparsity *α* indicates which components the target signals have, that are also corresponding to the atoms *d_k_* in the dictionary *D*. Therefore, for a qualified dictionary learning model, the learning dictionary *D* should cope with the sparsity *α* well. Based on this idea, an alternating optimization is applied to determine the dictionary *D* in the training stage, and K-SVD algorithm is allocated to solve the problem [37].

(3) $$\underset{D,\alpha }{\min}\left\{{\left\|x-D\cdot \alpha \right\|}_{F}^{2}+\lambda g_{\alpha }\left(\alpha \right)\right\}$$

where ||•||*_F_* is the *F*-norm calculation, *λ* is the regularization parameter, and *g*_•_(•) is the unified sparsity measurement function. Note that diverse *F*-norms act different roles in dictionary learning. *F* = 0 specifies the number of non-zero elements, by which the sparsity dominates, but the solution is usually NP-hard. *F* = 1 indicates Manhattan Distance, which enables sparse weights and feature extraction. *F* = 2 represents Euclidean Distance, which could simplify the model and prevent overfitting. In addition to the computing features, the different distance measures also reveal diverse physical properties of NILM problems, which is worthy of further exploration and research. In this work, the most commonly used Euclidean Distance is utilized for the dictionary model.

Once the dictionary *D* is determined after training, it becomes a straightforward process to identify the component in testing stage, i.e.,

(4) $$\alpha =\underset{\alpha }{\arg\min}\left\{{\left\|x-D\cdot \alpha \right\|}_{F}^{2}+\lambda g_{\alpha }\left(\alpha \right)\right\}$$

#### 3.1.2. Multiple Solution Scoring under Greedy Mechanism

The basic dictionary learning formulation in (4) only provides a certain solution to a given problem. However, since the dictionary learning belongs to the greedy algorithm, it has the capability to provide optimal solution set. For this goal, a sequential optimization approach is proposed based on dictionary learning model.

(5) $$\begin{array}{l} \alpha_{1}=\underset{\alpha_{1}}{\arg\min}\left\{{\left\|x-D\cdot \alpha_{1}\right\|}_{F}^{2}+\lambda g_{\alpha }\left(\alpha_{1}\right)\right\}\\ \alpha_{2}=\underset{\alpha_{2},\alpha_{2}\neq \alpha_{1}}{\arg\min}\left\{{\left\|x-D\cdot \alpha_{2}\right\|}_{F}^{2}+\lambda g_{\alpha }\left(\alpha_{2}\right)\right\}\\ \alpha_{3}=\underset{\alpha_{3},\alpha_{3}\notin \left\{\alpha_{1},\alpha_{2}\right\}}{\arg\min}\left\{{\left\|x-D\cdot \alpha_{3}\right\|}_{F}^{2}+\lambda g_{\alpha }\left(\alpha_{3}\right)\right\}\\ \vdots \\ \alpha_{C}=\underset{\alpha_{C},\alpha_{C}\notin \left\{\alpha_{1},\alpha_{2},\alpha_{3},...\right\}}{\arg\min}\left\{{\left\|x-D\cdot \alpha_{C}\right\|}_{F}^{2}+\lambda g_{\alpha }\left(\alpha_{C}\right)\right\} \end{array}$$

where *α*_1_, *α*_2_, *α*_3_,…, *α_C_* are the candidate solutions for individual classifiers.

As seen, by sequential approach, candidate solutions act different roles in this classifier. Because in the combined classifier the candidate solutions from diverse classifiers would be compared together, a fair evaluation approach is required. Hence, a probabilistic evaluation scoring based on object distance measures is proposed to fairly evaluate each solution in each individual classifier.

(6) $$\begin{array}{l} \mathrm{score}\left(\alpha_{1}\right)=100\\ \mathrm{score}\left(\alpha_{i}\right)=\left\{\begin{array}{c} Score,\mathrm{if}\;Score>0\\ 0,\mathrm{if}\;Score\leq 0 \end{array}\right.,i=2,3,\ldots ,C\\ Score=100\times \left(1-r_{p}\times \frac{{\left\|x-D\cdot \alpha_{i}\right\|}_{F}^{2}-{\left\|x-D\cdot \alpha_{1}\right\|}_{F}^{2}+\lambda \left(g_{\alpha }\left(\alpha_{i}\right)-g_{\alpha }\left(\alpha_{1}\right)\right)}{{\left\|x-D\cdot \alpha_{1}\right\|}_{F}^{2}+\lambda g_{\alpha }\left(\alpha_{1}\right)}\right) \end{array}$$

where *r_p_* is the regulation parameter for the scoring.

Following above calculation, the candidate solutions from individual classifiers are labelled with scores, and diverse classifiers are comparable due to the standardization of the relative computation. Therefore, a probability distance scoring system is established for fair evaluation.

#### 3.1.3. Feature Selection Based Classifier Deployment

To NILM problem, the measured signals can be technically categorized into real power, reactive power, and current harmonics. And different appliances show distinct behaviors on diverse features. For example, the motor-driven appliances show high demand for reactive power, and appliances belonging to disparate types have unique current harmonics [38]. Hence, diverse electric features have diverse recognition capability for different appliances, so the classifiers are mutually distinct when the bagging data are reorganized based on feature selection.

The illustrated formulations here are based on above considerations, i.e., the individual classifiers are formulated based on diverse electric feature selections. Specifically, we abandon one of the measured features at each time and form personalized classifiers under bagging framework. Since the real power is extremely important in load disaggregation, this feature is reserved for all classifiers.

(7) $$\underset{\alpha }{\min}\left\{{\left\|\mathrm{norm}\left(P\right)-D_{P}\cdot \alpha \right\|}_{F}^{2}+\sum_{*\in LS}\lambda_{*}{\left\|\mathrm{norm}\left(*\right)-D_{*}\cdot \alpha \right\|}_{F}^{2}+\lambda g_{\alpha }\left(\alpha \right)\right\}$$

where norm(⋅) is the normalization function. D_*_ is the dictionary for the normalized electric feature of *. λ_*_ is the regularization parameter for electric feature of *. *LS* is the load signature features apart from real power *P*. For example, if real power *P*, reactive power *Q*, fundamental current *H*1, third harmonic *H*3 and fifth harmonic *H*5 are considered, then *∈{*Q*, *H*1, *H*3, *H*5}. And during bagging stage, we form four individual classifiers with the selective electric features, i.e., {*P*, *Q*, *H*1, *H*3} for classifier 1, {*P*, *Q*, *H*1, *H*5} for classifier 2, {*P*, *Q*, *H*3, *H*5} for classifier 3, and {*P*, *H*1, *H*3, *H*5} for classifier 4.

By making full use of NILM characteristics, the deployed classifiers are efficient in ensemble method theoretically. Besides, by introducing the independent individual classifiers, the problem is scalable, depending on the individual classifier numbers in the bagging.

### 3.2. Two-Stage Decision-Making for Combined Classifier

**Figure 2:**
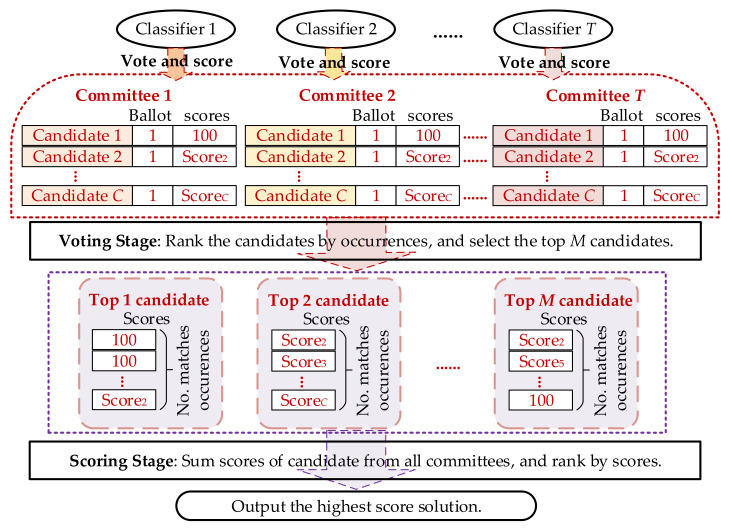
Two-stage decision-making process for combined classifier.

The decision-making process is visualized in Figure 2, where all candidate solutions from individual classifiers are rolling into the mixed solution pools for the two-stage campaign.

#### 3.2.1. Voting for Preliminary Evaluation

As shown in Figure 2, in the proposed strategy each individual classifier acts as a committee, and vote for every candidate generated by itself. If one candidate appeals in multiple committees, we count for the occurrence number regardless of the sequence in the solution pool. Then this counting index is used to select the top *M* candidates for the second stage evaluation.

Since voting idea is commonly used for the prediction in classification problem, it is well proved to be an effective evaluation approach. In the proposed stage, only occurrence is concerned, which matches the voting idea well.

#### 3.2.2. Standardized Scoring for Final Decision

As mentioned above in Equation (6) and Figure 2, the individual classifier scores each solution in a standardized way. Following two items are considered for the establishment of standardization. Firstly, the optimal solution in each classifier, i.e., the first candidate solution *α*_1_ in Equation (5), is labelled with full score. So the highest score in every classifier is determined. Secondly, use the objective function value of proposed dictionary model to evaluate the other solutions’ scores. Here the objective of candidate solution *α*_1_ is set as the reference as shown in Equation (6). Therefore, the scores from diverse classifiers are comparable.

The scores number of top candidates exactly corresponds to the occurrences of this solution in voting stage. The final decision strategy is to add up all the scores for the top *M* candidates, and rank the highest candidate as the output solution.

The standardized scoring evaluation is essentially based on the probabilistic distance measures calculation. So, the probability characteristics are featured in the whole method.

### 3.3. Algorithm Flow for Ensemble Method Based NILM

**Figure 3:**
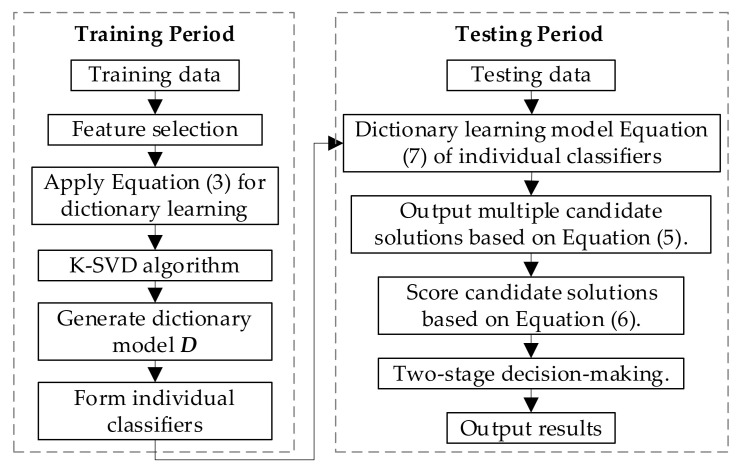
Algorithm flow of the ensemble method-based NILM.

Based on the deployed approaches for classifiers, the detailed algorithm flow of the ensemble method based NILM is shown in Figure 3. The algorithm flow can be divided into two periods, i.e., training and testing. During training period, the main object is to find the optimal configurations for each classifier, i.e., the parameters of each dictionary learning model. During testing period, it is a straightforward process to conduct the load disaggregation. Fine-tuning technology can be applied in two-stage decision-making block [39], e.g., on the parameter *r_p_* in Equation (6), to improve the overall performance.

## 4. Results and Discussions

The evaluation metrics are provided first in this section, followed by case studies based on simulations and field measurements.

### 4.1. Evaluation Metrics

Consider appliance s∈Ω_s_, where Ω_s_ is the set of all electrical appliances, then following definitions are denoted.

- True positive disaggregation *TP_s_*, denoting the number of results that are correctly detected as *s*.
- False positive disaggregation *FP_s_*, denoting the number of results that are incorrectly detected as *s*.
- False negative disaggregation *FN_s_*, denoting the number of results related to *s* that are incorrectly detected as other appliances.

Based on above definitions, the most widely applied three evaluation metrics in NILM can be calculated [39].

(8) $$P_{s}=TP_{s}/\left(TP_{s}+FP_{s}\right)\times 100\%$$

(9) $$S_{s}=TP_{s}/\left(TP_{s}+FN_{s}\right)\times 100\%$$

(10) $$F_{s}=\left(2\times P_{s}\times S_{s}\right)/\left(P_{s}+S_{s}\right)\times 100\%$$

where *P_s_* is precision metric for appliance *s*, *S_s_* is sensitivity metric for appliance *s*, and *F_s_* is *F*-measure metric for appliance *s*.

### 4.2. Verifications on Low Voltage Network Simulator

Firstly, Low Voltage Network Simulator (LVNS) is utilized for the comprehensive validation of our study. The LVNS platform is developed by research team from Power Disturbance & Signaling Research Laboratory (PDS Lab) in University of Alberta, focusing on the rigorous simulation of electrical networks spread from low voltage distribution to household consumption in North America. The rated real power and power factor of specific appliance are required as the input of the platform. As to the current harmonics, LVNS has initialized the harmonic current spectrums for each appliance, based on the typical harmonic current of the corresponding appliances. After the power flow calculations, the simulator could output the results of real power, reactive power and selected harmonics at the resolution of one snapshot per second. The detailed information about LVNS can be found in [40]. Since providing an adequate environment for NILM studies is one of the original motivations to develop the platform, it is very suitable for our research.

A typical North American residential house with almost twenty appliances is simulated in our study. The basic information of the simulated appliances can be found in Table 1. Other features, such as harmonic spectrums and usage patterns, are all following the settings in the platform [40]. Hence, the daily uses of each appliance are all uncertain under Monte Carlo framework. Multiple days’ simulations are conducted for the overall metrics and disaggregation performance analysis.

The proposed ensemble approach is denoted as *PEA* in the following discussions. The four individual classifiers, generated from the formula of Equation (7), are respectively denoted as *ICA*1, *ICA*2, *ICA*3, and *ICA*4 in this subsection. In detail, the load signature features utilized in the four individual classifiers are as {*P*, *Q*, *H*1, *H*3} for *ICA*1, {*P*, *Q*, *H*1, *H*5} for *ICA*2, {*P*, *Q*, *H*3, *H*5} for *ICA*3, and {*P*, *H*1, *H*3, *H*5} for *ICA*4. Besides, two traditional load disaggregation approaches are compared. The conventional optimization-based approach, inspired by the idea from [17], is denoted as *COA*. The traditional dictionary learning in [33], with all features considered at once, is also investigated in this study and denoted as *TDA*.

#### 4.2.1. Overall NILM Performance and Comparisons

By a statistical simulation test on the typical house, the general load disaggregation results in average metrics are shown in Table 2 and Table 3. As seen in Table 2, the conventional optimization-based approach is applicable in NILM problem, whose metrics are all over 80%. By introducing the sparsity measures in problem formulation, the load disaggregation is better formulated and the dictionary learning-based approach shows an enhancement. By utilizing ensemble strategy, the proposed approach outperforms all compared traditional approaches, and it is the only method whose *F*-measure is over 90%. Besides, the enhancements of the three metrics compared with *COA* are all over 5%, indicating the effectiveness of the research design. Since the data used in *COA*, *TDA* and *PEA* are the same, such results demonstrate the effectiveness of our ensemble strategy to improve the NILM performance with given dataset.

As seen in Table 3, following the feature selection-based bagging strategy, the individual classifiers are distinctive with each other. Among them, *ICA*2 with features of {*P*, *Q*, *H*1, *H*5} performs best, while *ICA*4 with features of {*P*, *H*1, *H*3, *H*5} performs worst. Such results indicate that the reactive power, fundamental current and fifth harmonic provide more featured load disaggregation information in the simulated case. Moreover, lack of reactive power as load signature feature may introduce a significant decline in performance, especially for the sensitivity metric. Comparing *ICA*1 with *ICA*2, it is observed that the usage of 5th harmonic current outperforms the usage of 3rd harmonic current, indicating the 5th harmonic is more effective in this case. Nevertheless, the proposed *PEA* outperforms all individual classifiers. To be specific, the ensemble strategy is effective in combining all individual classifiers, while the remarkable progress can be obtained for precision, sensitivity and *F*-measure. Especially for the sensitivity metric, the largest enhancement is over 10%, while the minimum increase is around 5%. Such improvement is noteworthy in NILM field. By deploying the proposed strategy, these weak classifiers are adequately combined together, forming a reliable and robust classifier for disaggregation.

In order to provide some insights of the load disaggregation performances by diverse approaches, the detailed results of average metrics for all simulated appliances are given in Table 4. As seen, the individual classifiers perform distinctively in load disaggregation, which makes the ensemble strategy effective in our study. For example, *ICA*1 is not credible in detecting FOO, while *ICA*4 comes across difficulties in recognizing CFL, CRTTV and LAP. Further on, *ICA*3 is totally ineffective in INC disaggregation, whose metrics are all zero. *ICA*2 is a relatively good method, and there is no obvious short board for all appliances. As to the harmonic selection discussions, we found 5th harmonic current outperforms 3rd harmonic in above general results, which is highly supported by appliance COF and FOO. Meanwhile, we also find that MW and LAP are more sensitive to the 3rd harmonic currents, indicating the diverse performance of different harmonics for different appliances. In general, these results demonstrate the efficiency of the proposed feature selection method for bagging and individual classifier training. As to the combined classifier, most of the ensemble decisions outperform the individual classifiers, referring to the effectiveness of the proposed two-stage decision-making process. However, there are some exceptions, e.g., COF for precision and FRZR for sensitivity. For these appliances the problem is that the metrics by combined classifier do not outperform the best individual classifier, but still achieves an acceptable value. Considering such scenarios do not affect the final performance of the ensemble strategy, the proposed method is proved to be effective in enhancing the robustness of NILM disaggregation.

As to the *TDA* that utilized all features at once, we found feature selection-based bagging strategy is effective in enhancing the NILM performance with identical data. This conclusion is followed by most appliances, so as to the general performance. *PEA* only underperforms *TDA* for INC, HEA and FRZR detections, but the gaps are extremely narrow. Another interesting phenomenon is also observed that *TDA* does not outperform individual classifiers for all appliances, such as STO, COF, FOO, etc. Such results indicate that in NILM problem, it is not always true that the more features, the better the disaggregation performance. The key is to utilize and combine the load features in a reasonable way.

#### 4.2.2. Performance of the Probabilistic Formulation in Ensemble Method

**Figure 4:**
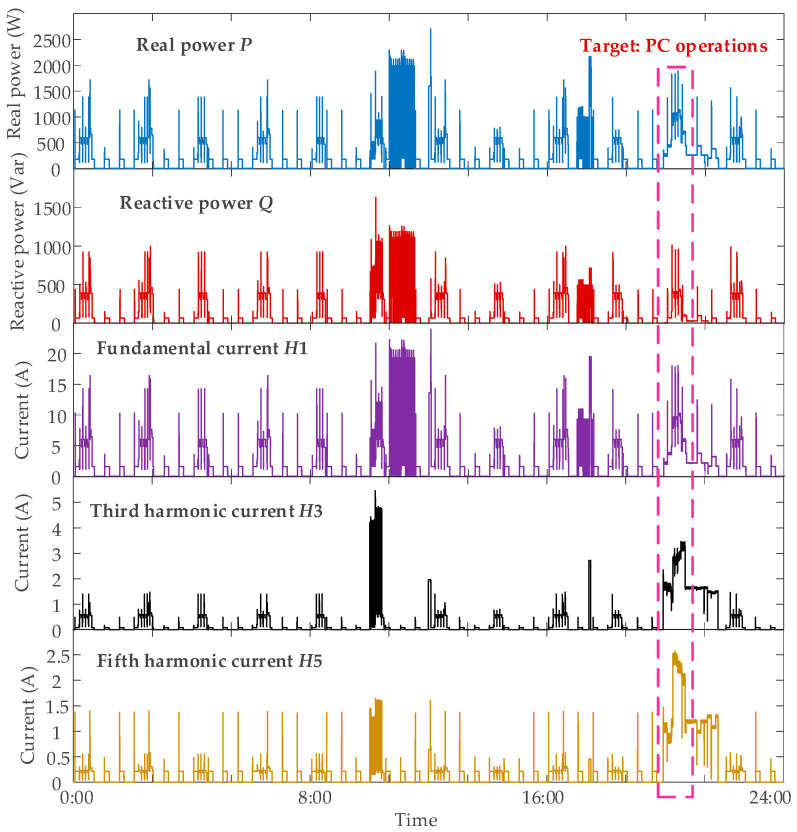
Detailed signal profiles of the measured data from phase A for certain day.

The discussions for the LVNS-based NILM are totally extended in this subsection, showing the roles of our proposed approaches in detail. A certain day is selected for the insight investigations, during which all the household appliances are operated. The signaling profiles of all electric features connected to phase A are illustrated in Figure 4, where the detections for appliance PC are discussed in the following part, acting as an example to reveal the contributions of our approach.

Note that the PC is a two-state appliance, whose operation power differs and fluctuates due to the power adapter. So, PC is a complex appliance in NILM problem, and effectively addressing this appliance would demonstrate the superiority of applied algorithm. This is the reason why PC is shown as an example here.

In the proposed four individual classifiers supported ensemble method, it is common and reasonable that if two classifiers provide true solution, the combined classifier is probably to output the true solution. However, in the period of PC operations, two interesting scenarios are found as shown in Table 5. In scenario 1, only *ICA*1 recognized PC correctly, and the other three classifiers all failed to tell the PC was ON. But the final decision outputs the true results successfully. In scenario 2, the first three classifiers missed the PC, while *ICA*4 considered PC was operating but in the wrong state. Even so, the proposed approach successfully outputs the right decision. Besides, traditional dictionary learning approach cannot detect PC correctly in both scenarios.

Results of scenarios in Table 5 fully support the rationality and contribution of our proposed probability framed method. Although the reasons behind two scenarios are similar, the vital points are different. Both scenarios are furtherly analyzed here.

**Figure 5:**
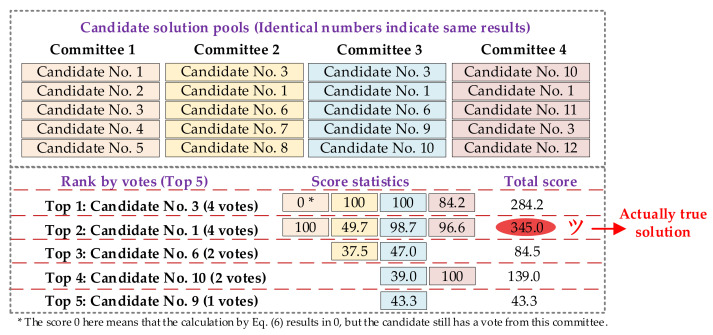
Detailed decision-making process for scenario 1.

The detailed decision-making process for scenario 1 is shown in Figure 5. As seen, if we do not introduce the probability framed multiple solutions as individual classifier outputs, the Candidate No. 3 will be determined as final decision by voting, which is not right. Besides, both candidate No. 3 and candidate No. 1 have four votes in the voting stage, so purely voting strategy has difficulty in determining which one is optimal. By applying the proposed scoring and two-stage decision-making process, the true solution is correctly recognized in this scenario.

**Figure 6:**
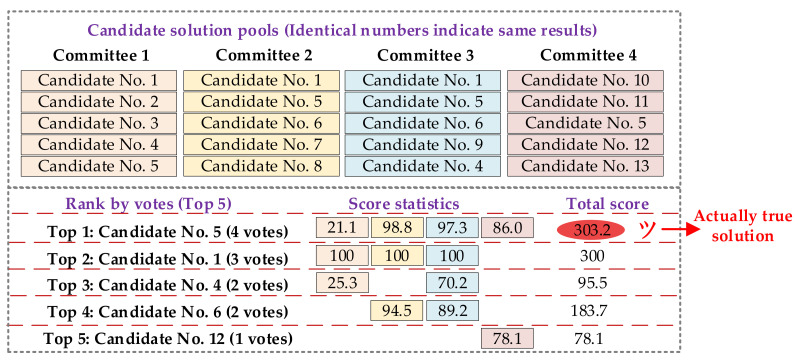
Detailed decision-making process for scenario 2.

The detailed decision-making process for scenario 2 is shown in Figure 6. Although the true solution can be selected by purely voting in this scenario, the single voting strategy shows infeasibility in scenario 1. Instead of directly voting, our proposed scoring also successfully recognizes the true solution, even when there are three individual classifiers recommending the same solution. Such severe situation can still be effectively addressed by the proposed method, showing the wide applicability of our method.

Combining two scenarios discussed above, the proposed probability framed ensemble framework is demonstrated to be non-redundant dealing with NILM problems. Considering the progress achieved in overall NILM performance, the proposed method is verified to be able to enhance the robustness of NILM in simulation environment.

### 4.3. Verifications on Field Measurements Dataset

A well-known field measurements-based public dataset called REDD [41], is used in our study for the validations in practical environments. REDD dataset was measured and collected by research team from MIT, and the original purpose was to provide field measurement data for energy disaggregation test. So this dataset is worldwide recognized and utilized in NILM verifications. There are six houses in the dataset, and House 1 is selected for our study. Since the reference operation states of appliances are only recorded in low frequency sampling, we only use multiple weeks of low frequency power data sampling at 1 Hz for our work. The detailed appliance information of House 1 can be found in Table 6.

Because low frequency data only contain the power information, the feature selection strategy for bagging is not available in this case. So, the original bootstrap sampling is used here for bagging, along with the fine-tuned regularization parameter *λ* for dictionary learning models. Still, four individual classifiers are formed following above strategy, and recorded as *ICA*1, *ICA*2, *ICA*3, and *ICA*4, respectively. The two traditional approaches discussed above are also investigated here, i.e., conventional optimization-based approach *COA* [17] and traditional dictionary learning-based approach *TDA* [33]. The proposed method *PEA* is compared with these approaches.

Table 7 provides the general results of REDD-based NILM for traditional approaches. As seen, due to the lack of load signature features, the load disaggregation performances of all approaches are not satisfied. Even so, there will be some discoveries. Firstly, the *COA* and *TDA* perform similarly, while the utilization of sparsity would increase the precision but decrease the sensitivity. Secondly, the proposed ensemble approach outperforms two traditional approaches, especially for the precision metric. Although only about 4% enhancement is seen for *F*-measure, it still demonstrates the effectiveness of our study under such data-poor situation.

Table 8 provides the general results of REDD-based NILM for individual classifiers. Comparing Table 8 with Table 7, we find there may be an enhancement for the precision and *F*-measure metrics by using bootstrap samples for training, but always a decrease for sensitivity. This may result from the insufficient data from the field measurements. By ensemble strategy, the precision is increased remarkably, resulting in around 85%. Such results demonstrate the effectiveness of our method dealing with field measurements with insufficient data. Besides, there is an increase for the overall evaluation metric *F*-measure, but the growth is slight. This is due to the limited enhancement for sensitivity metric. By only utilizing the real power for load disaggregation, the average sensitivities for all individual classifiers are all below 50%, which is hard to contribute to ensemble strategy. Nevertheless, in terms of overall performance, probability framed ensemble method is still effective in field NILM, even lacking of sufficient data.

Similarly, the detailed disaggregation metrics for all appliances are illustrated in Table 9. As seen, although general performance of the four individual classifiers is different, there are many identical or similar detection results for specific appliances, indicating the lack of diversity for individual classifiers. Even so, the enhancement has been observed for many appliances by introducing ensemble strategy, showing the effectiveness and flexibility of our method. Especially, we see a large increase in precision metric for appliance GFI and MW, while the results from individual classifiers are not reliable. Such results benefit from the random selection mechanism during the decision-making stage in ensemble method. By expanding the candidate solutions and introducing the probability evaluation, the NILM featured ensemble approach is better suited to load disaggregation for field measurements.

## 5. Conclusions

In this paper, the non-intrusive load disaggregation problem is thoroughly investigated from the view of ensemble method. A general NILM-oriented ensemble framework is established at the first stage, where both the individual classifiers and combined classifier are featured by probability model. The probability scoring strategy is proposed based on the probabilistic distance measures. Furthermore, the bagging strategy is customized for individual classifier constructions by feature selection method, which is proved to be effective under multi-feature acquisition systems. A two-stage decision-making process is allocated in combined classifier, taking into account the principles and characteristics of both ensemble strategy and load disaggregation. As an example of implementation, the dictionary learning-based approach is comprehensively modelled and investigated in our study, where both low voltage network simulator platform and field measurement dataset are utilized for verifications. Results show that by ensemble strategy, the NILM performance can be improved robustly. Besides, the proposed feature selection is effective, while the traditional bagging strategy is also efficient when lacking of sufficient input data, demonstrating the versatility and flexibility of the proposed method.

Therefore, the probability model framed ensemble method provides a new train of thought and a valid solution for the NILM problem, which is highly expected in both academic research and practical applications. Take this research as a foundation, the future works include, but are not limited to, the following aspects.

- Selection of individual classifiers. Bagging strategy is used in the presented ensemble framework, and feature selection is proposed based on the characteristics of NILM. However, other ensemble strategies, e.g., boosting and stacking, may also be effective and worth exploring, especially considering the problem features of NILM.
- Selection of combined classifier. A two-stage decision-making process for the combined classifier is investigated in this study. Although proved to be effective, the efficiency is related to the individual load disaggregation models. So explorations of a valid decision model for all scenarios are valuable.
- New probability models to cascade the framework. The distance measures based on the objective function of load disaggregation model are utilized for the probabilistic scoring in current work, whose performance is also related to the individual classifier model. If there is a universal scoring model for probabilistic evaluation, it will highly extend the scope and means of the ensemble method-based NILM.
- Potentials to cooperate with transfer learning. Non-intrusive detection of new appliances, or in new houses, is still a great challenge in NILM field. Only a few studies, majorly applying transfer learning or deep learning, have reported some achievements for these problems. Since ensemble methods naturally have multiple classifiers, it is feasible to realize new appliance or new house monitoring by appropriately deploying the classifiers with transfer learning idea. This research idea may initiate a new trend for the practicability investigations of NILM supported large-scale smart meter applications.
- Design with consideration of computation power for practical applications. The idea of generating multiple candidates from individual classifiers requires multiple optimization calculations, especially for greedy algorithms, which requires computing power in practical applications. Therefore, the proper design considering the computing resources and efficiency is an important concern toward practical NILM implementations.

## Figures and Tables

**Table 1:** The basic information of simulated appliances.

Operation Patterns	Appliances	Phase	Rated Power (W)	Power Factor	Code
Simple ON-OFF	Compact Fluorescent Lamp	A	60	0.9	CFL
	Food Processor	A	1600	1	FOO
	Incandescent Lamp	B	40	1	INC
	Microwave Oven	A	1200	0.99	MW
	Toaster	B	860	1	TOA
Repetitive ON-OFF	Coffee Maker	B	920	1	COF
	Regular Dryer	AB	4000	0.88	DRY
	Heater	B	1400	0.97	HEA
	Stove	AB	2000	0.9	STO
	ASD-based Washer	A	320	0.45	WSH
Repetitive ON-OFF with start transient	Freezer	B	220	0.9	FRZR
	Regular Fridge	A	180	0.94	RFR
Multi-state	LCD Television	B	300	0.99	LCDTV
	CRT Television	A	200	1	CRTTV
	Desktop PC	A	260	1	PC
	LCD Computer Monitor	B	160	0.96	LCD
	Laptop	B	75	0.96	LAP
Repetitive multi-state with transients	Furnace	A	600	0.84	FUR

**Table 2:** Results comparison of LVNS-based NILM for traditional approaches.

Metrics in Average Value	*COA*	*TDA*	*PEA*
*P_s_* (%)	89.83	94.02	94.99
*S_s_* (%)	82.56	85.07	87.14
*F_s_* (%)	85.19	88.30	90.23

**Table 3:** Results comparison of LVNS-based NILM for individual classifiers.

Metrics in Average Value	*ICA*1	*ICA*2	*ICA*3	*ICA*4	*PEA*
*P_s_* (%)	91.18	93.34	89.16	89.04	94.99
*S_s_* (%)	81.36	81.87	82.85	75.62	87.14
*F_s_* (%)	84.33	86.15	85.40	79.33	90.23

**Table 4:** Detailed results comparison of LVNS-based NILM (Appliance Level).

Metrics in Average Value		*ICA*1	*ICA*2	*ICA*3	*ICA*4	*TDA*	*PEA*
*P_s_* (%)	INC	99.10	99.50	0.00	97.95	94.59	99.74
	STO	86.73	87.06	88.79	89.23	81.28	89.53
	COF	69.78	99.62	84.93	76.80	83.07	79.55
	FOO	65.64	98.49	98.50	96.91	99.97	98.50
	TOA	83.08	80.31	90.99	90.43	87.64	89.93
	MW	99.95	93.44	98.09	96.65	98.71	97.94
	HEA	99.93	99.77	99.33	99.97	97.42	99.98
	CFL	97.69	99.92	97.03	84.50	98.79	98.62
	WSH	99.66	99.66	99.66	99.66	99.78	99.66
	DRY	99.88	99.85	99.88	99.95	99.57	99.96
	FRZR	99.99	99.99	99.99	95.70	99.95	99.99
	RFR	94.72	96.25	94.74	94.39	93.14	93.87
	LCDTV	86.15	87.45	87.19	79.22	84.67	87.00
	CRTTV	82.61	81.27	88.47	93.81	92.19	92.40
	PC	85.53	74.68	91.47	62.80	95.63	94.32
	LCD	99.96	99.96	100.00	99.97	100.00	99.48
	LAP	97.00	92.30	92.96	49.31	93.82	94.73
	FUR	93.86	90.69	92.88	95.46	92.07	94.71
	Average Value	91.18	93.34	89.16	89.04	94.02	94.99
*S_s_* (%)	INC	74.34	74.37	0.00	57.52	82.78	77.66
	STO	89.21	93.64	93.52	93.49	97.18	93.52
	COF	88.52	89.58	95.82	90.60	79.31	95.53
	FOO	27.90	86.83	91.51	91.13	84.49	90.60
	TOA	84.60	84.36	83.98	92.63	95.85	92.49
	MW	87.89	67.52	99.40	99.68	94.68	99.40
	HEA	54.15	49.38	89.68	77.47	85.09	80.69
	CFL	66.24	66.59	68.70	34.03	62.43	67.14
	WSH	96.42	96.42	96.42	96.42	90.09	96.42
	DRY	99.86	99.18	99.86	98.64	98.57	99.86
	FRZR	93.23	95.49	94.35	85.45	96.43	91.14
	RFR	85.88	79.15	80.83	73.13	75.21	81.52
	LCDTV	98.66	98.60	98.64	65.32	98.83	98.60
	CRTTV	79.93	66.88	70.30	31.04	66.17	70.92
	PC	59.30	64.98	64.57	63.48	57.99	66.75
	LCD	94.85	89.46	91.42	91.86	91.81	93.55
	LAP	86.67	74.58	75.76	26.36	75.84	75.74
	FUR	96.82	96.58	96.61	92.89	98.47	96.92
	Average Value	81.36	81.87	82.85	75.62	85.07	87.14
*F_s_* (%)	INC	84.16	84.38	0.00	67.77	88.21	86.63
	STO	88.14	90.65	91.51	91.73	87.77	91.89
	COF	73.86	93.76	88.73	80.45	79.30	85.36
	FOO	36.89	91.40	94.59	93.73	91.10	94.01
	TOA	83.61	80.77	87.00	91.49	91.09	91.19
	MW	93.23	76.70	98.74	98.09	96.61	98.65
	HEA	70.06	65.92	94.09	87.29	90.60	89.30
	CFL	78.85	79.73	80.28	46.06	76.23	79.76
	WSH	98.01	98.01	98.01	98.01	94.61	98.01
	DRY	99.87	99.51	99.87	99.28	99.06	99.91
	FRZR	96.41	97.69	97.02	89.83	98.15	95.23
	RFR	89.87	86.68	86.93	82.11	83.02	87.02
	LCDTV	92.49	93.19	93.06	69.27	91.01	92.94
	CRTTV	79.69	73.10	78.35	46.23	76.75	80.14
	PC	69.10	68.89	75.54	62.56	71.28	77.98
	LCD	97.31	94.34	95.47	95.68	95.68	96.15
	LAP	91.17	82.48	83.42	34.24	83.77	84.09
	FUR	95.29	93.54	94.68	94.15	95.13	95.79
	Average Value	84.33	86.15	85.40	79.33	88.30	90.23

**Table 5:** Two special scenarios for the PC related load disaggregation.

	*PEA*	*ICA*1	*ICA*2	*ICA*3	*ICA*4	*TDA*
Scenario 1	√ ^1^	√	× ^2^	×	×	×
Scenario 2	√	×	×	×	o ^3^	×

^1^ √: PC is operating, and also correctly detected. ^2^ ×: PC is operating, but falsely detected as OFF. ^3^ o: PC is operating in a certain state, but detected as operating in the other state.

**Table 6:** The basic information of studied appliances in House 1 of REDD.

Operation Patterns	Appliances	Rated Power (W)	Code
Simple ON-OFF	Lighting 1	70	LIG1
	Lighting 2	80	LIG2
	Lighting 3	60	LIG3
Simple ON-OFF with fluctuation or transients	Microwave Oven	1600	MW
	Bathroom GFI	1600	GFI
	Kitchen Outlet 1	1080	KO1
Repetitive ON-OFF	Washer	600	WSH
Repetitive ON-OFF with fluctuation or transients	Dryer ^1^	5400	DRY
	Kitchen Outlet 2	1540	KO2
	Oven 1	1650	OV1
	Oven 2	2500	OV2
	Regular Fridge	200	RFR
Multi-state with complicated modes and transients	Dish Washer	1000	DW

^1^ Dryer is connected on phase A-B.

**Table 7:** General results comparison of REDD-based NILM for traditional approaches.

Metrics in Average Value	*COA*	*TDA*	*PEA*
*P_s_* (%)	57.68	60.49	84.37
*S_s_* (%)	53.51	49.17	49.62
*F_s_* (%)	49.44	49.62	53.99

**Table 8:** General results comparison of REDD-based NILM for individual classifiers.

Metrics in Average Value	*ICA*1	*ICA*2	*ICA*3	*ICA*4	*PEA*
*P_s_* (%)	60.15	70.52	70.52	62.90	84.37
*S_s_* (%)	47.55	47.75	48.00	44.07	49.62
*F_s_* (%)	48.61	50.44	50.61	45.66	53.99

**Table 9:** Detailed results comparison of REDD-based NILM (Appliance Level).

Metrics		*ICA*1	*ICA*2	*ICA*3	*ICA*4	*TDA*	*PEA*
*P_s_* (%)	DRY	91.40	92.14	92.14	92.14	91.40	92.14
	GFI	15.18	15.21	15.21	15.82	13.53	95.24
	KO1	0.00	0.00	0.00	0.00	0.00	0.00
	KO2	98.25	99.01	99.01	99.01	99.23	99.01
	LIG1	97.70	98.04	98.04	98.04	99.66	98.04
	LIG2	98.52	98.52	98.52	98.52	99.72	98.52
	LIG3	0.00	99.90	99.90	0.00	0.00	99.90
	MW	0.00	0.00	0.00	0.00	0.00	100.00
	OV1	16.96	15.78	15.78	15.96	16.96	15.78
	OV2	81.79	99.50	99.50	99.50	82.20	99.50
	RFR	99.25	99.40	99.40	99.40	99.81	99.40
	WSH	83.28	99.60	99.60	99.60	83.61	99.60
	DW	99.66	99.66	99.66	99.66	99.66	99.66
	Average Value	60.15	70.52	70.52	62.90	60.49	84.37
*S_s_* (%)	DRY	89.00	98.10	98.10	98.10	89.00	98.10
	GFI	17.52	13.12	13.12	13.12	16.37	14.06
	KO1	0.00	0.00	0.00	0.00	0.00	0.00
	KO2	45.97	45.97	45.97	45.97	45.07	45.97
	LIG1	72.46	67.96	71.27	59.23	94.19	71.27
	LIG2	14.52	14.51	14.51	14.39	14.52	14.51
	LIG3	0.00	38.98	38.98	0.00	0.00	38.98
	MW	0.00	0.00	0.00	0.00	0.00	20.13
	OV1	70.95	83.73	83.73	83.73	70.95	83.73
	OV2	96.04	96.04	96.04	96.04	96.52	96.04
	RFR	91.67	81.38	81.38	81.38	92.23	81.38
	WSH	41.76	19.06	19.06	19.06	41.93	19.06
	DW	78.21	61.89	61.89	61.89	78.47	61.89
	Average Value	47.55	47.75	48.00	44.07	49.17	49.62
*F_s_* (%)	DRY	90.18	95.03	95.03	95.03	90.18	95.03
	GFI	16.27	14.09	14.09	14.34	14.81	24.50
	KO1	0.00	0.00	0.00	0.00	0.00	0.00
	KO2	62.64	62.79	62.79	62.79	61.99	62.79
	LIG1	83.21	80.27	82.54	73.85	96.85	82.54
	LIG2	25.31	25.29	25.29	25.12	25.36	25.29
	LIG3	0.00	56.08	56.08	0.00	0.00	56.08
	MW	0.00	0.00	0.00	0.00	0.00	33.51
	OV1	27.38	26.55	26.55	26.81	27.38	26.55
	OV2	88.35	97.74	97.74	97.74	88.79	97.74
	RFR	95.31	89.49	89.49	89.49	95.87	89.49
	WSH	55.63	32.00	32.00	32.00	55.85	32.00
	DW	87.64	76.36	76.36	76.36	87.94	76.36
	Average Value	48.61	50.44	50.61	45.66	49.62	53.99

## Data Availability

The data presented in this study involve simulation data and public dataset. The simulation platform is available in reference [40] and the public dataset is openly available in reference [41].
